# Identification of Reference Genes for Expression Studies in the Whole-Blood from Three Cattle Breeds under Two States of Livestock Weather Safety

**DOI:** 10.3390/ani11113073

**Published:** 2021-10-28

**Authors:** Kelly J. Lozano-Villegas, Roy Rodríguez-Hernández, María P. Herrera-Sánchez, Heinner F. Uribe-García, Juan S. Naranjo-Gómez, Rafael J. Otero-Arroyo, Iang S. Rondón-Barragán

**Affiliations:** 1 Immunobiology and Pathogenesis Research Group, Faculty of Veterinary Medicine and Zootechnics, University of Tolima, Altos the Santa Helena, A.A 546, Ibagué 730006299, Tolima, Colombia; kjlozanov@ut.edu.co (K.J.L.-V.); mpherreras@ut.edu.co (M.P.H.-S.); hfuribeg@ut.edu.co (H.F.U.-G.); jsnaranjogo@ut.edu.co (J.S.N.-G.); 2 Poultry Research Group, Laboratory of Immunology and Molecular Biology, Faculty of Veterinary Medicine and Zootechnics, University of Tolima, Altos the Santa Helena, A.A 546, Ibagué 730006299, Tolima, Colombia; royrodriguezh@ut.edu.co; 3 Grupo de Investigación en Reproducción y Mejoramiento Genético Animal, Facultad de Ciencias Agropecuarias, Universidad de Sucre, Sincelejo 700001, Sucre, Colombia; rafael.otero@unisucre.edu.co; 4 Laboratorio de Reproducción Animal, Corporación de Ciencias Biotecnológicas, Embriotecno, Montería 230029, Córdoba, Colombia

**Keywords:** cattle, farm animals, environmental conditions, heat stress, mRNA, genes normalization, real-time PCR

## Abstract

**Simple Summary:**

Reductions in the fertility, body weight, and growth rate of cattle across the world are associated with the global warming phenomenon. Developing optimal management strategies is an important aspect of breeding programs for different breeds. Blood tissue undergoes dramatic physiological and metabolic changes during heat stress conditions, which involves the expression and regulation of a great number of genes across species. Real-time quantitative PCR (qPCR) is a method for the rapid and reliable quantification of mRNA transcription. Reference genes are used to normalize mRNA levels between different samples. Thus, the selection of high-quality reference genes is necessary for the interpretation of data generated by real-time PCR.

**Abstract:**

Real-time PCR is widely used to study the relative abundance of mRNA due to its specificity, sensitivity, and repeatability quantification. However, relative quantification requires a reference gene, which should be stable in its expression, showing lower variation by experimental conditions or tissues. The aim of this study was to evaluate the stability of the expression of five commonly used reference genes *(actb*, *ywhaz*, *b2m*, *sdha*, and *18s rRNA*) at different physiological stages (alert and emergency) in three different cattle breeds. In this study, five genes (*actb*, *ywhaz*, *b2m*, *sdha*, and *18s rRNA*) were selected as candidate reference genes for expression studies in the whole blood from three cattle breeds (Romosinuano, Gyr, and Brahman) under heat stress conditions. The transcription stability of the candidate reference genes was evaluated using geNorm and NormFinder. The results showed that *actb*, *18SrRNA*, and *b2m* expression were the most stable reference genes for whole blood of Gyr and Brahman breeds under two states of livestock weather safety (alert and emergency). Meanwhile, *actb*, *b2m*, and *ywhaz* were the most stable reference genes for the Romosinuano breed.

## 1. Introduction

Heat stress is a physiological condition that occurs when an animal cannot dissipate body heat, leading to an increase in body temperature [1]. In livestock production, the heat stress decreases body weight, average daily gain, growth rate, fat thickness, meat quality, and milk production [2]. Cattle exposed to high temperatures also exhibit alterations in folliculogenesis and oocyte viability [3]. Additionally, heat stress decreases pregnancy rates and embryonic development in embryos produced in vivo and in vitro [4]. Due to heat stress effects, humans have reevaluated management decisions regarding which animals to use for food production [5]. In this way, breeds that originated in warm climates such as African zebu (*Bos primigenius indicus*) and African taurus (*Bos taurus africanus*) show adaptive advantages to heat stress compared with breeds that originated in temperate areas such as European taurus (*Bos taurus taurus*) [5,6].

The heat stress in cows can be evaluated through the change in behavior and physiological variables such as respiratory rate, heart rate, and vasodilation [7]. Furthermore, the quantification of gene expression for conserved proteins that increase their expression under heat stress conditions allows them to be used as a reference to evaluate the stress of an individual [8]. The qPCR technique allows the quantification of gene transcript expression [9]. Relative quantification requires a reference gene, which should be stable in its expression and show lower variation by experimental conditions or tissues [10]. Initially, highly conserved genes that code for proteins involved in functional processes and the structure of cells were chosen, which were previously called housekeeping genes [11]. The use of these reference genes for qPCR data normalization may have solved problems that could affect the quantification, such as the concentration variability of RNA and inhibitors from the extraction protocols [12]. Likewise, the use of reference genes as endogenous controls in the relative quantification can allow the correction of the sample variations [13]. However, it has shown that the gene expression can be variable in some experimental conditions, and it has been necessary to validate the stability of these genes in different conditions [14].

In cattle, the expression stability of several references genes such as actin beta—*actb*, glyceraldehyde-3-phosphate dehydrogenase—*gapdh*, succinate dehydrogenase—*sdha*, tyrosine 3-monooxygenase/tryptophan 5-monooxygenase activation protein zeta—*ywhaz*, TATA-box binding protein—*TBP*, beta-D-glucuronidase—*GUSB*, H2A clustered histone 14—*H2AC14*-, peptidylprolyl isomerase A—*PPIA*, ribosomal protein L15—*RPL15*, battenin—*CLN3*, eukaryotic translation initiation factor 3 subunit K—*EIF3K* in the bovine liver, kidney, pituitary gland, thyroid gland, muscle, and mammary gland have been reported [15,16]. In livestock production, despite the use of temperature and humidity index in the control of heat stress in cattle, the appropriate reference genes for cattle under heat stress conditions are still clear. Due to qualities such as accessible source of systemic information of the transcriptome that allows measuring changes in relevant biological processes and pathways, blood samples are considered good samples [17]. In the present paper, the expression stability of five reference genes (actb, ywhaz, b2m, sdha, and 18S rRNA) in whole blood from Romosinuano, Gyr, and Brahman cattle breeds collected under two states of livestock weather safety (alert and emergency) were evaluated.

## 2. Materials and Methods

### 2.1. Ethics Statement

All procedures involving animals were approved by the Ethics committee of the University of Tolima based on the Law 84/1989 and the Resolution 8430/1993 and complied with the guidelines for animal care and use in research and teaching [18,19].

### 2.2. Study Population

Healthy cows of Brahman (*n* = 10), Gyr (*n* = 10), and Romosinuano (*n* = 10) breeds (age between 48 and 96 months) were located on a farm near to Monteria city, Cordoba department at northern region of Colombia, (Latitude 8°45′36″ N and Longitude 75°53′08″ W), between April and November of 2020, with an average temperature of 29 °C and relative humidity between 70 and 85%.

### 2.3. Weather Data

Ambient temperature (°C) and relative humidity measured as a percentage for each hour throughout the study was measured using a PCE-FWS20N weather station (PCE Instruments™, Meschede, Germany). The temperature-humidity index (THI) was calculated for each hour applying the National Research Council (1971) formula as follows:

(1) THI=(1.8×Tdb+32)−[(0.55–0.0055×RH)×(1.8×Tdb−26)

THI data were used to identify two categories of livestock weather safety index (alert and emergency) [20]. In our study period, an alert condition period was identified from 21:00 to 08:00 h with THI values of 75 to 78, and an emergency state was identified from 13:00 to 14:00 h with THI values of 84 to 86.1. Therefore, the blood samples for the gene expression analysis were taken at 7:00 h with a THI value of 76.3 (alert state) and 14:00 h with a THI value of 86.1 (emergency state).

### 2.4. Samples, RNA Extraction, and cDNA Synthesis

Blood samples were obtained by venipuncture of the caudalis medium vein, transferred into 4 mL EDTA tubes (Becton Dickinson Vacutainer Systems, Franklin Lakes, NJ, USA), and collected twice daily at 7:00 h and 14:00 h. Immediately after sample collection, blood samples were divided into small volume aliquots of 2  mL in Graduated Safelock Microcentrifuge Tubes. Later, all blood samples were frozen in liquid nitrogen and stored at −20 °C until experimental analysis.

RNA was extracted from blood samples using the RNA-solv reagent kit (OMEGA, Norcross, GA, USA) according to the manufacturer’s protocol with certain modifications. The modified RNA extraction protocol consisted of 1000 μL of RNA-Solv^®^ reagent (OMEGA, Norcross, GA, USA), which was mixed with 200 μL of whole blood. The mixture (sample and RNA-Solv^®^ reagent) was homogenized in a vortex (30 s); then, 200 μL of chloroform (J.T.Baker^®^, Radnor, PA, USA) at −20 °C were added, vortexed (30 s), and incubated at 4 °C for 5 min. The mixture was centrifuged at 12,000 rpm for 15 min at 4 °C, and the aqueous phase was transferred to a clean tube. For the precipitation stage, 2 volumes of isopropanol were added to the recovered aqueous phase and mixed by inversion (6 times) followed by incubation at 4 °C for 30 min. Later, centrifugation was performed at 12,000 rpm for 10 min at 4 °C to obtain a pellet, which was washed twice as follows: 1 mL of 75% ethanol (Merck, Darmstadt, Germany), centrifugation at 12,000 rpm during 10 min at 4 °C, and discarding the supernatant. Finally, the pellet was dried for 5 min at room temperature and dissolved in DEPC water (21 μL); afterwards, RNA quality was measured by spectrophotometry with the NanoDrop One (Thermo Scientific, Wilmington, DE, USA), and the pellet was stored at −20 °C.

Prior to reverse transcription, all RNA samples were diluted to 200 ng/μL, and cDNA was synthesized using GoScript^TM^ Reverse Transcription System kit (Promega, Madison, WI, USA) following the manufacturer’s instructions. End-point PCR and agarose gel electrophoresis were conducted to determine the cDNA quality and the amplicon size.

### 2.5. Gene Selection and Primer Design

Five reference genes, *actb*, *18S rRNA*, *b2m*, *ywhaz*, and *sdha* were selected as candidate reference genes for this study based on previous reports [15,16]. Primers were designed based on sequences from *Bos taurus* and *Bos indicus* using Geneious Prime software v2021.1 [21] (Table 1).

### 2.6. End-Point PCR and Quantitative Polymerase Chain Reaction (qPCR)

All primers were examined for their target specificity by end-point PCR with a total volume of 25 µL, composed of 14.8 µL of distilled–deionized water, 5 µL of 5X green GoTaq^®^ Flexi Buffer (Promega, Madison, WI, USA), 1 µL of dNTPs (1.5 mM) (Invitrogen, Carlsbad, CA, USA), 1 µL of each primer (forward and reverse) (10 pmol/µL), 1 µL MgCl_2_ (25 mM), 0.125 µL of GoTaq^®^ Flexi DNA polymerase (Promega, Madison, WI, USA), and 1 µL of the cDNA as template. The amplification was carried out in a ProFlex^TM^ PCR System (Applied Biosystems, Carlsbad, CA, USA) with an initial denaturation step at 95 °C for 3 min, which was followed by 35 cycles of denaturation at 95 °C for 30 s, annealing at the specific annealing temperature for each set of primers (Table 1) for 30 s, extension at 72 °C for 30 s, and a last step of final extension at 72 °C for 5 min. Amplicons were revealed on 1% agarose gel by electrophoresis (PowerPac™ HC, Bio-Rad, Hercules, CA, USA) using a GeneRuler 100 bp DNA Ladder (Thermo Fisher Scientific, Waltham, MA, USA). The gel was stained with HydraGreen™ (ACTGene, Piscataway, NJ, USA) and visualized under UV light, using the ENDURO^TM^ GDS gel documentation system (Labnet International, Inc., Woodbridge, NJ, USA).

Relative gene expression of *b2m*, *sdha*, *ywhaz*, *actb*, and *18S rRNA* genes was measured by qPCR using a Luna^®^ Universal qPCR Master Mix (New England BioLabs Inc., Beverly, MA, USA) in a QuantStudio 3 Real-Time PCR System (Thermo Fisher Scientific, Waltham, MA, USA), by the Fast ramp program. Thermal cycling conditions were initial denaturation 1 min at 95 °C; then, 40 cycles of denaturation for 15 s at 95 °C and annealing for 30 s at 60 °C. Subsequently, a melting step was performed at 95 °C for 1 s, 60 °C for 20 s, and a continuous rise in temperature to 95 °C at a rate of 0.15 °C per second. Each sample was run in triplicate.

### 2.7. Analysis of Reference Gene Expression Stability

Expression levels of the tested reference genes were quantified by the quantification cycle (Cq) values obtained through qPCR from the three technical replicates, averaged, and used as input data on NormFinder and geNorm to evaluate the gene expression stability [14,23,24].

## 3. Results

### 3.1. Primer Specificity

**Figure 1:**
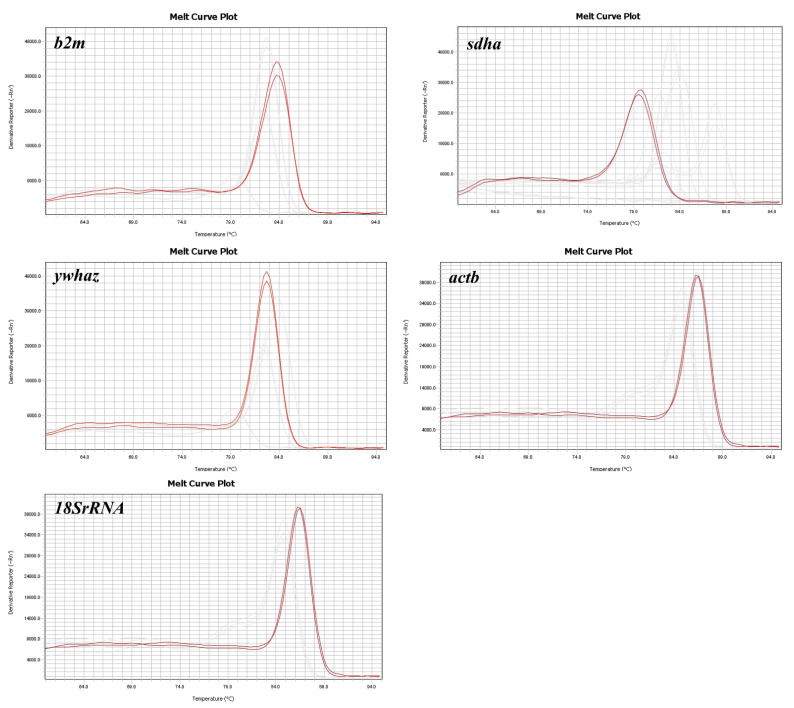
Melting curve of *b2m*, *sdha*, *ywhaz*, *actb*, and *18SrRNA* gens in whole blood of cattle.

Five reference genes for *Bos* species were chosen for this study based on previous reports [15,16] (Table 1). All the primers designed for the reference genes were specific through evaluation by end-point PCR and qPCR; as shown in Figure 1, the qPCR melting curves showed a single peak, suggesting that there was no formation of primer dimers or nonspecific PCR products.

### 3.2. Expression Profiles of Reference Genes

**Figure 2:**
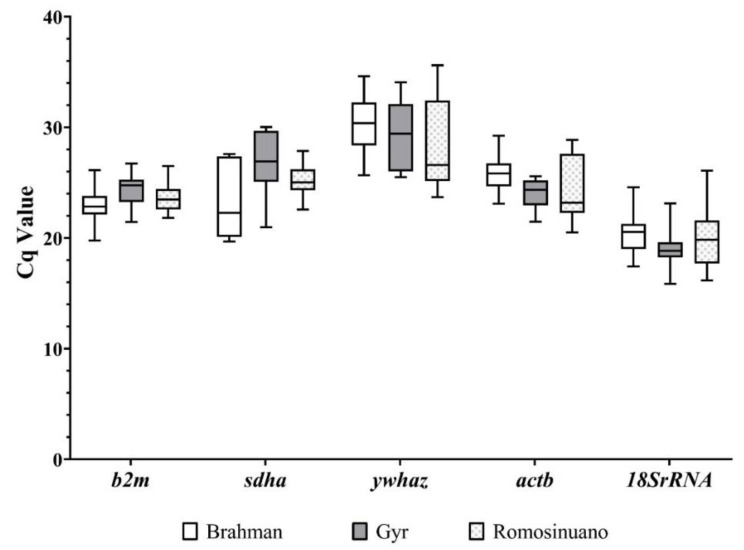
Cq values for five reference genes of the blood samples among different bovine breeds. The Cq values of the *b2m*, *sdha*, *ywhaz*, *actb*, and *18S rRNA* reference genes from Brahman (white boxes), Gyr (dark gray boxes), and Romosinuano (dotted light gray boxes). The box indicates the 25th and 75th percentiles, the lines represent the median, squares represent the means, and whiskers represent the maximum and minimum values.

As shown in Figure 2, the Cq values of the five reference genes from blood samples among Brahman, Gyr, and Romosinuano breeds ranged between 15.86 and 35.61. *18SrRNA* was the most highly expressed gene, with Cq values ranging between 15.86 and 26.8, followed by *b2m*, *sdha*, and *actb*, which showed Cq values from 19.77 to 26.72, 19.68 to 30.03, and from 20.50 to 29.23, respectively. In addition, *ywhaz* exhibited Cq values from 25.50 to 35.61 (Figure 2).

### 3.3. Reference Gene Stability: geNorm

**Figure 3:**
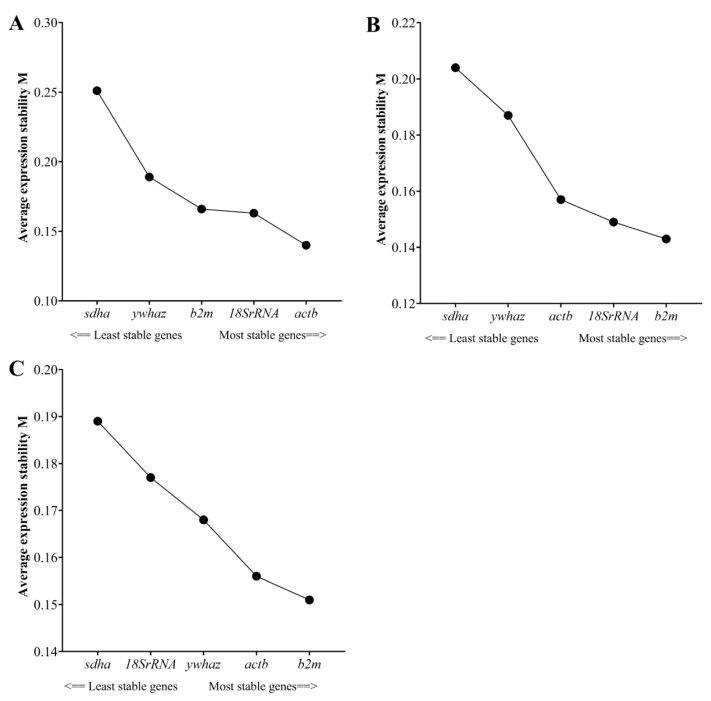
Transcriptional stability value of five candidate reference genes calculated by the geNorm algorithm. The average expression stability value (M) was calculated for *actb*, *18SrRNA*, *b2m*, *ywhaz*, and *sdha* genes on different bovine breeds: Brahman (**A**), Gyr (**B**), and Romosinuano (**C**).

The expression stability of the reference genes in terms of M values was analyzed using geNorm software. As shown in Figure 3, the stability ranking of the five reference genes was different among bovine breeds. However, all reference genes had an M value below 1.5, which is the recommended geNorm (the most stable reference genes have the lowest M values), and the *b2m* gene was the most stable gene (Figure 3).

### 3.4. Reference Gene Stability: NormFinder

The reference gene stability value was calculated for each gene using NormFinder software, indicating that those with the lowest stability values are the most stable genes. NormFinder identified *actb* and *b2m* as the two most stable genes with stability values of 0.016 and 0.021 respectively, in contrast with *sdha* gene (Table 2) with values of 0.029 to 0.043.

## 4. Discussion

The real-time PCR is a powerful tool for evaluating mRNA levels due to its specificity, sensitivity, and repeatability quantification [25,26,27]. However, when the expression of the target gene is analyzed by this method, there are unavoidable operational errors; e.g., in the absolute expression level, the same target gene can display significant errors between different biological groups or technical repetitions [28]. This is unlike relative quantification, where the RNA transcription level is normalized based on the expression level of the internal reference gene [29]. The ideal reference gene should be stably expressed, and its expression should not be affected by the experimental conditions [30]. Numerous studies have demonstrated that the expression of commonly used reference genes varies among different cell types, tissues, and experimental conditions; for example, *actb* and *gapdh*, which are largely accepted, can show large variations in expression [31,32]. Thus, the selection and validation of reliable reference genes for each particular condition are essential to quantitative accuracy [33].

Several studies have been conducted to assess the reference genes in specific tissues in numerous species [34]. In cattle, De Ketelaere et al. (2006) selected *sdha*, *ywhaz*, and *18S rRNA* as being the most stable genes for the accurate normalization of qPCR of bovine polymorphonuclear leukocytes [35]. Likewise, *sdha* has also been ranked greatest in terms of expression stability in bovine neutrophils [36]. However, the reference genes mentioned previously were described for different experimental conditions. In the present study, two statistical methods (geNorm and NormFinder) were used to evaluate the gene expression stability of five reference genes (*actb*, *ywhaz*, *b2m*, *sdha*, *18SrRNA*) in the whole blood of three cattle breeds under two states of livestock weather safety. The Temperature–Humidity Index has been widely used to alert cattle producers of potential weather-based heat stress; for example, some recommendations for mitigating heat stress are based on estimating THI values [20,37,38]. In the present study, two states of heat stress (alert THI = 70–80 and emergency THI ≥ 84) in cattle were chosen for blood sample collection due to the expected cellular stress responses in these states [5,17,37,39].

The geNorm and NormFinder software were used to evaluate the stability of the reference genes. The geNorm method calculates the gene stability value (M) by computing pairwise comparisons and geometric averaging of each reference gene under different experimental conditions, where genes with the smallest M values below 1.5 are considered excellent constitutive genes [14]. On the other hand, the NormFinder method assesses gene expression stability (Stability Value, SV) based on parameters of the estimates for both intragroup and intergroup variations of each gene [23]. Based on the geNorm program, the most stable reference genes were *actb*, *18SrRNA*, and *b2m* for Brahman, and those for the Gyr breeds were *b2m*, *18SrRNA,* and *actb.* Whereas for Romosinuano, *b2m*, *actb*, and *ywhaz* were the most stable genes (Figure 3). The stability ranking of the reference genes presented here is consistent with previous studies [40,41] According to the stability ranking, *b2m* is considered a good reference gene for emergency conditions, and these data agree with several studies that have suggested *b2m* be one of the reliable reference genes under different experimental conditions [42,43,44].

NormFinder identified *actb* as the most stable gene for Brahman and Romosinuano breeds, while for Gyr, *18SrRNA* was the most stable gene (Table 2). Genes such as *actb* and *18SrRNA* have been successfully used as reference genes in other studies [45,46,47]. *actb* gene has been widely used as an internal control for different experimental assessments due to this gene encoding one of the six existing actin proteins, which are involved in cell motility, structure, and integrity, which is essential for all cellular physiological conditions [1,48]. Regarding the *18srRNA* gene, it is widely used as an internal control gene for normalization in gene expression because it has a low turnover rate and is less prone to substantial changes due to physiological disturbances [49]. In this study, *sdha* was the least stable gene in all of the three cattle breeds using two statistical methods. Nevertheless, it has been used as a reference gene in other studies [35,50].

The current Minimum Information for Publication of Quantitative Real-Time PCR Experiments (MIQE) suggests the use of more than one reference gene in all qPCR studies [51]. Following MIQE and despite the discrepancy in the ranking orders of reference genes observed by different software (geNorm and NormFinder), *actb*, *18srRNA*, and *b2m* were consistently identified as the most stable reference genes for the Brahman breed, and *actb*, *b2m*, and *ywhaz* were the most stables genes for the Romosinuano breed. Regarding the Gyr breed, the most stables genes were *b2m*, *18srRNA*, *actb*, and *ywhaz*. The reference genes differences between breeds can be explained by the genetic diversity of the cattle breeds shaped by evolutionary forces such as genetic drift, migration, selection, and geographical separation [52]. *Bos indicus* of Indian origin and *Bos taurus* of European and African origin are the two main cattle subspecies [53]. In general, *Bos indicus* cattle breeds (Brahman and Gyr) have a greater adaptive capacity to stressful environments than *Bos taurus* breeds [54]. In tropical countries, *Bos indicus* breeds such as Gyr and Brahman are very important because of its tolerance of heat and parasites and because they are essential to the breeding of hybrids [55].

Notwithstanding, some *Bos taurus* breeds adapted to tropical climates might be heat tolerant and exhibit a higher reproduction, growth, and carcass quality than *Bos indicus* breeds [53]. For example, the Romosinuano tropically adapted *Bos taurus* is a breed native to Colombia, South America, that is characterized by having a high reproductive efficiency [56,57]. In this way, differences in the reference genes can be linked to the genetic difference related to the subspecies and breed differences.

## 5. Conclusions

In conclusion, by using two statistical methods to determine the expression stability of five reference genes under heat stress conditions, our study suggests the use of the geometric mean of *actb*, *18srRNA*, and *b2m* genes (for Gyr and Brahman) and *actb*, *b2m*, and *ywhaz* genes (for Romosinuano) as suitable reference genes for the normalization of gene expression.

## Figures and Tables

**Table 1:** Primers of candidate reference genes.

Gene	Primer Sequence (5′–3′)		Primer Length (nt)	Tm (°C)	GC%	Annealing Temperature (°C)	Amplicon Size (bp)	Reference
*b2m*	F	CTGCTATGTGTATGGGTTCC	20	55.6	50	54	141	[22]
	R	GGAGTGAACTCAGCGTG	17	54.8	58.8			
*sdha*	F	TGCAGACCATCTACGGAGCGGA	22	65.44	59.09	55	163	This study
	R	ACGTAGGAGAGCGTGTGCTTCCTCC	25	67.96	60.00			
*ywhaz*	F	AGCAGGCTGAGCGATATGAT	20	59.02	50.00	55	180	This study
	R	TCTCAGCACCTTCCGTCTTT	20	58.95	50.00			
*actb*	F	GGGATGAGGCTCAGAGCAAGAGA	23	63.65	56.52	60	118	This study
	R	AGCTCGTTGTAGAAGGTGTGGTGCC	25	66.91	56.00			
*18S rRNA*	F	TAGAGGGACAAGTGGCGTTC	20	59.39	55.00	55	104	This study
	R	CGCTGAGCCAGTCAGTGTAG	20	60.46	60.00			

**Table 2:** Reference gene stability value ranked by NormFinder algorithm in different bovine breeds.

Ranking	Brahman		Gyr		Romosinuano	
	Gene ^1^	Stability Value	Gene ^1^	Stability Value	Gene ^1^	Stability Value
1	*actb*	0.009	*18SrRNA*	0.009	*actb*	0.017
2	*18SrRNA*	0.018	*b2m*	0.016	*b2m*	0.017
3	*b2m*	0.021	*ywhaz*	0.018	*ywhaz*	0.020
4	*ywhaz*	0.025	*actb*	0.021	*18SrRNA*	0.022
5	*sdha*	0.043	*sdha*	0.032	*sdha*	0.029

^1^ Putative reference genes are listed from top to bottom in order of decreasing stability value for each of the three breeds.

## Data Availability

Not applicable.
